# 

**Published:** 1900-02-10

**Authors:** 


					TffBlHOSPITAL.' ^ ?? The standard of highest purity.|'?Lancet. February 10th, 1900-
n
OADcf,y.RY's "t H B'!CAC^HPY'S
COOOA ^  ^ H y NO OKUQ8 OR ALKALIES USBO.
ABSOLUTELY PUKE. * "
No. 698>* Vol. XXVII. [Sifl Saturday,
Feb. 10, 1900.]
[Subscription Terms,] pRICE TWOPENCE.

				

